# Why Copper Amalgam Sometimes Wastes in the Mouth

**Published:** 1891-05

**Authors:** W. B. Ames

**Affiliations:** Chicago, Ill.


### Why Copper Amalgam Sometimes Wastes in the Mouth.

BY W. B. AMES, D.D.S., CHICAGO, ILL.

Read before the Mississippi Valley Dental Society, at Cincinnati, March, 1891.

Inasmuch as many of us have for several years been endeavoring to attain to some definite knowledge of the physical properties and peculiarities of amalgamated copper, and as my convictions derived from an experience of ten years in the use, and four years in the manufacture of the material are somewhat at variance with those of some gentlemen who have written and spoken on the subject, I have concluded to bring it before this society for consideration.

The most serious shortcoming that has been urged against copper amalgam as an offset to its many valuable characteristics, is the tendency in many cases to waste or cup out, and it has seemed to many to be a sufficiently serious objection to cause its abandonment, or to make it necessary to first make a test filling to discover whether the use of this material was warranted in the mouth of the patient in hand. My faith in the material has never been shattered, for the reason that, although some of my most conscientious efforts in the making and use of it have resulted in more or less dismal failure, I have had the conviction that the difficulty was to be attributed to the faulty processes and methods of making and using rather than to inseparable peculiarities. This conviction was backed by having used some time since a quantity of copper amalgam called Sullivan's, made for the Dental Mfg. Co., London, which differs materially from the "Sullivan's" furnished by Ash & Sons. This amalgam I found to stand in mouths in which all other preparations of the kind would waste to a greater or less extent, and furnished me the incentive to labor toward ascertaining the cause of the lack of stability of other copper amalgams. That the wasting was most serious in what is termed acid mouths was not a very tangible clue to start upon, yet it was most natural to consider first what solvents of the constituents of copper amalgams we might naturally have within the mouth. It would be unreasonable to suppose that there was ever a condition of the saliva sufficiently acid to dissolve copper and mercury to the extent that we often see, unless the action was in some way intensified, as these metals are only soluble in nitric acid and the more powerfully nitro-muriatic, unless the less energetic acids be used in connection with a galvanic couplet, *i. e.*, as the fluid of a battery of which one of these metals is the positive element or as the electrolyte with one of the metals in question as the anode. In the case of copper there might be a very slight wasting from oxidation and sulphuretting of the surface, these compounds being either dissolved or worn from the surface so that there might be a continual reformation, but in the case of the mercury which is the metal which is supposed to be presented upon the surface, there could be no such wasting, as it does not oxidize under the conditions present, and the sulphuret which is the compound formed in a cold state, would be a very tenacious and insoluble film. The black film which the fillings take on, which do not show any evidence of wear is undoubtedly the black sulphuret of mercury —that being the compound that is formed when mercury and sulphur are brought together in the cold state.

The only tangible clue to the solution of copper amalgam in the mouth seems to be the fact that outside of the mouth the galvanic current would cause the solution of its components in the weak acids, and the phenomena observed in the mouth, in the more rapid wasting of those fillings which were so placed as to form the positive element of a battery, the negative element of which was an adjoining or occluding gold filling or crown.

Starting with this as a clue, it was natural to take notice of the fact that many of the copper amalgams when made at all dry presented the appearance of being composed of copper amalgam and free copper, the surface showing in some specimens almost a copper-colored surface, and it was most natural to question whether or not this free copper was the positive element and the amalgamated portion the negative element of a galvanic battery when placed in the fluids of some mouths. This would readily account for the entire phenomena, for it would be analagous to the solution of impure, unamalgamated zinc in dilute sulphuric acid where the same zinc with an amalgamated surface is not dissolved. Upon treating with a solution of mercurious nitrate amalgams containing free copper, it was found that the nature of the material was radically changed, a much more thorough amalgamation being brought about, and with it much better results being obtained in the mouth.

Now, what is the cause of this free copper in copper amalgam? With that of my own manufacture it was largely due to an extensive grinding of the amalgamated copper crystals in a mill devised for the purpose, with a view of giving it smoothness and a more desirable plasticity. It has been the general opinion, I think, that copper amalgam could not be triturated too extensively by the manufacturer or by the dentist at the time of using. In several published descriptions of methods of producing the material great stress has been placed on the heating and rubbing down, and repeating this, until sufficient mercury had been worked out and smoothness obtained. On account of results observed in using amalgam made by myself by a large variety of processes, decided some time since that all, or any grinding was bad for the material, and that the only heat that it should have, was that given to it by the dentist at the time of its preparation for the filling. When the amalgamation of the copper is obtained by precipitating it upon the surface of a mass of mercury by the use of electricity, as I originally made the material, the first crystallization takes place with a large surplus of mercury, so that heat is absolutely necessary to put it in marketable condition, and unless the copper has been precipitated in a uniformly fine state the grinding must be resorted to, whereas, if the copper is precipitated upon some other than a mercury surface and the amalgamation of a proper quantity effected by the use of proper chemicals, the excess of mercury can be worked out and the material put into marketable condition without heat and without grinding. That heating, and especially repeated heating, is bad for copper amalgam where the very best results are required is unquestionable, as the 250° F. that is required to break up the crystals and set mercury free, is sufficient to volatilize the mercury to a very perceptible extent, as can be seen by holding a piece of gold over the amalgam during the process. While I do not consider careful heating to be as injurious as the extensive trituration that has been so generally advised, I think that it is well to use only fresh amalgam in such cases as we have reason to fear that wasting might take place.

With copper amalgam in which the copper precipitate has been carefully amalgamated, and the amalgamation has not been disturbed by heat or grinding, we have a material that will have permanency and stability in the most acid mouth.

Where a filling has wasted, it has in nearly all cases a surface to which fresh amalgam will readily attach, so that these fillings can be easily filled out, and if this is done with an amalgam that has the proper stability, the filling is practically as good as if the wasting had not taken place.

DISCUSSION.

The discussion of Dr. Ames' paper was opened by Dr. L. E. Custer, Dayton, O.

The general explanation of the cause of the waste of amalgamated copper fillings has been that it was due to an acid condition of the saliva—that saliva of an acid reaction would dissolve these fillings. 1st. If the saliva is acid of sufficient strength to dissolve the copper, or mercury of a copper amalgam filling, it would not only attack the enamel of the teeth but it would, in like manner act upon the silver, tin, zinc, platinum or gold, or whatever metals may compose an ordinary alloyed amalgam filling, some of which are more easily soluble than copper. A common amalgam filling does not disappear from this cause. 2nd. If copper amalgam fillings are dissolved by acid they should waste pretty equally all over their surface, which in all cases they do not. 3rd. Some copper amalgam fillings waste while others in the same mouth do not. This would not occur if it is due to an acid saliva. 4th. If copper amalgam is susceptible at all to the action of acids, as solvents, they should waste between the teeth where acids, the product of fermentation of food particles, are generated. And finally, copper amalgams sometimes waste in an alkaline saliva as well as one of acid reaction.

The fact is, gentlemen, that saliva of an acid reaction is not the direct cause of the waste of copper amalgam fillings, but is simply a condition which favors the waste of certain kinds of copper amalgams which characterized the first attempts at its manufacture in this country. Because the waste of some copper amalgams occurred in an acid saliva, it is no proof that this fluid is a solvent; but by this coincidence we are led to the belief that there is in some way a connection between the two.

In order to understand what this relation is we must go down to first principles, as in the paper just read. We must first know what copper amalgam is; what changes it is likely to undergo in the mouth, and under what conditions it would most likely lose substance.

This material consists of copper particles in a fine state of division whose surfaces are more or less amalgamated. If this amalgamation is perfect there will be no copper surface exposed at all, yet if one of these particles be broken in two its whole inside will present a clean copper surface. We must understand that there is no fusion of one metal into the other. In a filling these metals are like the bricks and mortar of a house. The mercury is represented by the mortar and the copper particles by the bricks.

Whether these copper particles are entirely amalgamated or only partially so is a very important thing to be taken into consideration. A second consideration is in regard to the proportion of mercury. There may be simply enough to thoroughly amalgamate the copper particles or there may be enough to float them about. A third consideration is in regard to the manner in which it is handled by the operator preparatory to filling.

These conditions are in regard to the material itself. Bu there are two other factors which come from without which must be considered; these are the saliva and attrition. All these conditions and influences must be borne in mind before we can approach the question at hand.

You are aware that we have been using a great variety of copper amalgams, all of which have given different results. Copper is a metal very difficult to amalgamate, and there is no less than four different methods of producing amalgamation, each of which produces a different article. Then again, after the copper is amalgamated every manufacturer does not extract the same amount of mercury and also every operator manipulates in a different manner preparatory to filling. The first copper amalgam made in this country was by the mercuric nitrate method and by precipitating with iron or zinc. This was often contaminated with other metals. It was coarse, not well amalgamated, often contained twice the necessary amount of mercury and was used with about as much wisdom and discretion as though it were hot mush, and no wonder the cry went up form Brooklyn that there would be a grand howl some day. The use of these different materials in the same mouths would give different results. And to go into the detail of the different materials and the different conditions and influences would require too much time; however, we may consider the effect of perfect amalgamation and its opposite, and the quantity of mercury upon the waste of this material under an alkaline or acid saliva and attrition.

A perfectly amalgamated filling presents as stated in the paper a pure mercury surface to the action of the saliva and attrition.

Saliva of an alkaline reaction would have no effect upon mercury, neither would it if it were acid of the strength found in the mouth since mercury is dissolved most easily only by strong nitric acid. The effect of attrition upon such a filling would probably be to dislodge the amalgamated copper particles. But since perfectly amalgamated copper can scarcely be touched by a file this too must be excluded. Therefore, if a perfectly amalgamated copper filling always presents a clean mercury surface it would stand in an acid mouth and under attrition. According to the paper mercury forms a salt in the mouth—the sulphuret—which, as Dr. Ames says, is insoluble, so this would not be effected by the saliva. But if this salt should be easily dislodged there would be a continual reformation at the expense of the filling, and such a one would waste when exposed to attrition. But as stated in the paper this forms a tenacious film. So we must agree with the conclusions arrived at by the author that a perfectly amalgamated copper filling properly used will not waste. If during the finishing of the filling at the second sitting the unamalgamated centres of the copper particles on the surface of the filling are exposed, in an acid saliva these would probably waste by the galvanic solution and the process will stop when these are dissolved; but if in an alkaline saliva, salts would form, these would be removed by attrition again and again until all those exposed particles have been converted into salts and removed.

A poorly amalgamated filling presents copper and mercury areas on the exposed surface of the filling. If the copper has not been fully amalgamated before filling it is hardly probably that it will become so afterwards, so that these two metals will remain separate and subject to the action of the saliva and attrition. On general principles we would exclude an alkaline saliva as having any effect upon copper. But if in an alkaline saliva the two should form salts and these are exposed to attrition it would depend entirely upon how strongly these are attached or whether these are insoluble, whether the filling wastes or not. The copper salts we know can even be removed with a tooth brush so that when such a force as attrition plays upon the filling these salts are sure to be removed, and the repeated loss and reformation of salts at the expense of the filling accounts for their waste in this position. Then the copper salts, and especially the sulphuret, are soluble in water so that they may be dissolved as fast as formed.

When we subject a poorly amalgamated filling to an acid saliva we have a very different result. We have all heard this paper which treated of such conditions. Where formerly the waste of such a filling was supposed to be due to chemical solution by an acid saliva, Dr. Ames explains this very satisfactorily as being due to galvanic solution. It will not be necessary to go into this any further than to add what he possibly intentionally omitted, and that is, that it is not necessary that a fluid be strongly acid to produce galvanism, so a saliva which simply has this reaction is capable of producing the phenomenon. Whereever this local galvanic action is going on the surfaces are fresh, which accounts for the bright appearance and which led to the former belief that it was chemical action rather than galvanic. In receding positions, if these poorly amalgamated surfaces should become protected by the formation of insoluble salts, the galvanic solution ceases at that point.

The proportion of mercury is an important consideration, and especially where the filling is to be subjected to the force of mastication. When the amalgamated copper particles touch one another on all sides and the inequalities are filled with mercury, I think we have the best material, and according as the proportion of the mercury is increased and the copper particles are separated from one another, will the strength be lessened and it will lose substance when exposed to attrition. The first amalgams made, contained a large amount of mercury and their waste in exposed positions was largely due to this fact.

It would be interesting to go into the therapeutics of the copper and mercury salts. If it is true, as stated in the paper, that those fillings do not waste from mercury salts, what will become of our beautiful theory of the effect of copper salts, unless those of mercury are soluble; for how can an insoluble salt be antiseptic? All these would be interesting, but are not in place here.

The practical points to be derived are these: Poorly amalgamated fillings should not be used in acid mouths, or in alkaline ones when exposed to attrition, but perfectly amalgamated fillings with no excess of mercury and properly manipulated, may be used in all positions and conditions of saliva and not waste.

Dr. J. S. Cassidy, Covington, Ky., attributes the wasting to imperfect amalgamation and perhaps galvanic action, there being no chemical union of the metals entering into the composition of the amalgam.

Dr. E. G. Betty, suggests finishing such fillings at times of insertion and not to touch them afterward. It has been his experience that if copper amalgam is washed with a nitrate of mercury solution before insertion and the filling subsequently turns black, it will wear well.

Dr. C. M. Wright, thinks copper amalgam the most uncertain of any of the filling materials that we possess. That while its antiseptic properties are perhaps beneficial, many of the difficulties of manipulation must be corrected to make it a success as a filling material.

Dr. J. Taft: Does not all the difficulty come from the feeble affinity of the two materials for each other? Is anything to be gained in the use of copper amalgam over ordinary amalgams? The materials in these have a strong affinity for each other while the affinity in copper amalgam is feeble, What more can we expect than the formation of salts and cupping of the filling? Until this affinity can somehow be increased it seems to me of but little value as a filling material.

Dr. W. B. Ames, Chicago, said he never had placed much dependence upon the antiseptic action of copper amalgam, for where there is no waste of the filling, the color of the filling next to the dentine remains light colored, while that in fillings that wash, is dark. Unless the antiseptic salts, indicated by the dark color, are formed we get no antiseptic action; so in fillings that do not waste there would be no antisepsis. He did not think the affinity in copper amalgam was so feeble as Dr. Taft supposes. While the copper is not permeated by mercury it is strongly coated with it and would require more heat than that in the mouth to dislodge the particles.

